# Serial Interval and Intervention Efficiency in Pertussis Outbreak, South Korea, 2024

**DOI:** 10.3201/eid3205.251304

**Published:** 2026-05

**Authors:** Andrei R. Akhmetzhanov, Bianca de Padua, Jonathan Dushoff

**Affiliations:** College of Public Health, National Taiwan University, Taipei, Taiwan (A.R. Akhmetzhanov, B. de Padua); McMaster University, Hamilton, Ontario, Canada (J. Dushoff)

**Keywords:** pertussis, whooping cough, respiratory infection, serial interval, epidemiological time intervals, bacteria, bacterial infection, South Korea

## Abstract

We estimated an unmitigated mean serial interval during a school-based pertussis outbreak in South Korea at 14.7 (95% credible interval 9.0–27.4) days, comparable with previous estimates. Public health interventions reduced the effective reproduction number by 65% (95% credible interval 26%–88%), which likely brought it to <1 and contributed to curbing the outbreak.

Pertussis cases have increased worldwide recently, driven by waning vaccine immunity, disruptions to vaccination programs, and a reduction in transmission during the COVID-19 pandemic (*1**,**2*). In addition, there is an increase in reports of *B. pertussis* strains resistant to macrolide antimicrobial drugs, a first-line treatment, in the Western Pacific region since 2008, and specifically in Japan during 2024–2025 (*3*). As of mid-2025, Japan reported a 10-fold increase in pertussis cases compared with 2024, and South Korea reported a >20-fold increase.

The natural history of pertussis infections remains unclear, and more accurate estimates of epidemiologic time intervals are urgently needed, especially the serial interval (SI), which is the time interval between symptom onset in those spreading the infection and those being infected. In a recent investigation of a school-based pertussis outbreak in South Korea in 2024 (*4*), the mean SI was estimated to be 9.5 days, which closely aligned with another recent report (*5*) but greatly differed from a previous estimate of 20.5 days from the Netherlands (*6*). Although differences in case definitions could contribute to this discrepancy, the estimated observed SI was affected by public health interventions such as case isolation. It is necessary to estimate a counterfactual unmitigated SI for the future planning of outbreak response, because it more accurately reflects the natural history of the pathogen.

To estimate the unmitigated SI, we extracted the data from the previously published report (*4*) and built a latent transmission model to account for the effect of intervention (Appendix). Data were publicly available in the original publication, and no specific permission was required. During the outbreak, March–June 2024, the major intervention on April 17, 2024, followed a test-trace-isolate approach with screening of all suspected cases (*4*). The isolation of confirmed cases and prophylactic measures provided to close contacts have a dual effect on the transmission dynamics; they shorten the SI and reduces the reproduction number by lowering the contact rate (*7*).

We then fitted the transmission-pair data with a model that incorporated 2 consequences of intervention: a shortening of the SI distribution for transmissions in the postintervention period compared with the preintervention period, and a reduction in the mean number of secondary cases generated by a primary case in the postintervention period. The effect of the intervention was quantified by a transmission-reduction parameter, 𝜀, which ranged from 0 (no effect of isolation) to 1 (no transmission after isolation). Following the parameters of the original study (*4*), we modeled the number of secondary cases with a negative binomial distribution. We assumed the overdispersion parameter was the same in both periods. We termed the resulting SI distribution (corresponding to preintervention dynamics) the unmitigated SI. We estimated the observed SI distribution for comparison when no effect of intervention was incorporated.

Statistical inference yielded a mean unmitigated SI of 14.7 days (95% credible interval [CrI] 9.0–27.4 days), a broader distribution than the observed SI (mean 10.4 days; 95% CrI 7.6–14.6 days) (Figure 1). The medians of both SIs were <10 days (9.9 days and 7.9 days); however, the unmitigated distribution had a much longer tail (95th percentile, 42.8 days). The unmitigated distribution we found is similar to that estimated in a study from the Netherlands (*6*), with the probability mass slightly shifted toward shorter time intervals. The Netherlands study recruited families of infants <6 months of age hospitalized with pertussis, potentially inducing selection bias toward more severe cases, which could explain the shift toward shorter intervals in our estimate.

**Figure 1:**
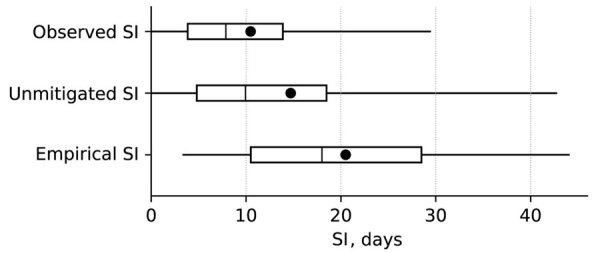
Comparison of estimated SI distributions from a school-based pertussis outbreak in South Korea, 2024. Observed SI from the original study on which we based our study (*4*) is shown compared to the unmitigated SI inferred after accounting for public health interventions and empirical SI from a household survey conducted in the Netherlands (*6*). Each boxplot displays the interquartile range (box tops and bottoms), 95% credible interval (whiskers), median (vertical line), and mean (solid circle). SI, serial interval.

We estimated a preintervention reproduction number of 1.4 (95% CrI 0.65–2.6) and an intervention efficiency of 65% (95% CrI 26%–88%), which represents the relative reduction in per case transmissibility after the intervention. The postintervention reproduction number was <1, at 0.45 (95% CrI 0.15–1.01). We illustrated the temporal variation in forward-looking reproduction number and mean generation time (mean SI) (*8*) (Figure 2).

**Figure 2:**
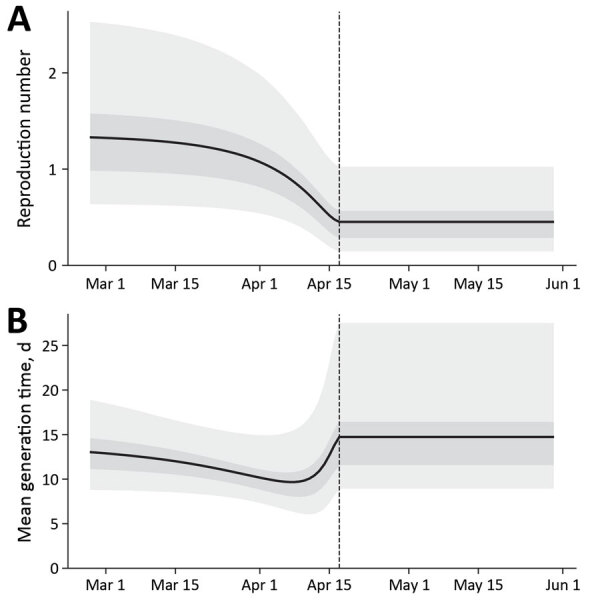
Temporal variation in the forward-looking reproduction number and the mean generation time across the outbreak period of a school-based pertussis outbreak in South Korea in 2024. A) Graph of the forward-looking reproduction number, defined as the average number of secondary infections generated by a case infected on a given date, accounting for interventions throughout their infectious period. B) Graph of the mean generation time, defined as the average interval between the infection of a primary case and the infections they cause. Vertical dashed line indicates the start of the main intervention (2024 Apr 17). Solid lines represent posterior means; light and dark shaded areas indicate 95% and 50% (interquartile) credible intervals.

Our findings suggest that isolation likely contributed to curbing the pertussis outbreak. The previous study mentioned that the delay between symptom onset and confirmation leading to case-isolation was ≈6 days on average and 25% of cases were confirmed after 20 days (*4*). Those results imply the isolation of cases was not immediate and that larger values of intervention efficiency could be achieved with shorter delays. Nevertheless, the effect of isolation cannot be disentangled from other supplementary interventions such as active case finding and targeted prevention for close contacts of case-patients. Because of the long unmitigated SI, a response relying solely on symptom-based isolation might be insufficient, which implies that rapid contact-tracing and preemptive testing are likely required to efficiently control future outbreaks.

Appendix Additional information about serial interval and intervention efficiency in pertussis outbreak, South Korea, 2024
