# Supplementary material for: Serial Interval and Intervention Efficiency in Pertussis Outbreak, South Korea, 2024
Source: Emerg Infect Dis. 2026 May;32(5):809–11. doi: 10.3201/eid3205.251304 (PMC13174972; doi:10.3201/eid3205.251304)
Supplement: Appendix — Additional information about serial interval and intervention efficiency in pertussis outbreak, South Korea, 2024 [file 25-1304-Techapp-s1.pdf]

Article DOI: https://doi.org/10.3201/eid3205.251304

EID cannot ensure accessibility for supplementary materials supplied by authors. Readers who have difficulty accessing supplementary content should contact the authors for assistance.

Serial Interval and Intervention Efficiency in Pertussis Outbreak, South Korea, 2024

Appendix

This Appendix describes the statistical framework for estimation of the generation time, serial interval, and reproduction number of pertussis from school-based outbreak data in South Korea, 2024. First, the incubation period was estimated using historical records and employing a Bayesian model that accounts for interval-censored observations. Three candidate distributions (gamma, Weibull, lognormal) were considered and the best-fit distribution was selected using a Bayesian mixture model (Section S1). This distribution was then used to backproject symptom onset dates to presumptive infection times for each case reported during the 2024 outbreak (Sections S2–S3). With these imputed infection times, the generation time distribution was inferred under two scenarios: (*i*) the observed distribution, reflecting the dual effect of the outbreak investigation of 17 April 2024 on per-case transmissibility and on the realized generation time; and (*ii*) the counterfactual unmitigated distribution, representing what would have been expected without intervention (Section S4). The intervention effect was modeled by a single parameter, ε, that simultaneously shortened the realized generation time and reduced the case reproduction number, following a framework previously developed by Chan and Nishiura ([13]). Similarly to the inference of the incubation period, the generation time distribution was modeled by three candidate distributions (gamma, Weibull, lognormal) with the best-fit distribution selected using a Bayesian mixture model (Section S4). Technical details on implementation and coding are provided in Section S6.

S1 Incubation period

S1.1 Historical data

To backproject cases from their symptom onset dates to presumptive exposure times, we first estimated the incubation period distribution for pertussis. Given scarcity in incubation

period estimates available in the literature (*1–4*), we used two historical sources for our analysis (*5*,*6*). Eleven of these records were from a 1936 study (*5*) and two were from a study on experimental pertussis infection (*6*). The aggregated data are shown in **Appendix Table**. In each instance, symptom onset was defined as the first signs of an upper respiratory infection (*4*). The incubation period of each case was recorded as a time interval in days, $[X_{m,L}, X_{m,R} + 1 \text{ day})$, where $X_{m,L}$ and $X_{m,R}$ are the left and right bounds, respectively ($m = 1, \ldots, 13$). For example, an incubation period recorded as "5 days" implies $X_{\circ,L} = X_{\circ,R} = 5$ days, meaning that the incubation period spans from the start of day 5 to the start of day 6. Similarly, an incubation period recorded as "less than seven days" implies that $X_{\cdot,L} = 0$ day and $X_{\cdot,R} = 6$ days, with the interval spanning from day 0 up to the start of day 7.

S1.2 Likelihood

Because the data were doubly interval-censored without any prior information on the timing of the events within their respective intervals, we assumed that both the symptom onset and exposure times were distributed uniformly within those intervals. This assumption adds uncertainty to the estimates rather than introducing systematic bias. Specifically, the symptom onset for each case occurring within a one-day interval was sampled as:

1. $s_m \sim \text{Uniform}\left(\text{lower} = X_{m,L},\ \text{upper} = X_{m,R} + 1 \text{ day}\right)$

The corresponding exposure time e_m was then sampled consistently with the observed incubation period interval $\left[X_{m,L}, X_{m,R} + 1 \text{ day}\right)$:

2. $e_m \sim \text{Uniform}\left(\text{lower} = 0 \text{ day},\ \text{upper} = \min\left(\{s_m, X_{m,R} - X_{m,L} + 1 \text{ day}\}\right)\right)$

where we also constrain the difference $s_m - e_m$ to be non-negative.

We fitted the incubation periods ($s_m - e_m$) to three candidate distributions: gamma, Weibull, and lognormal. To select the best-fitting model, we used a Bayesian mixture model framework (*7*). This approach assigns a weight to each distribution. For computational efficiency, we specified the mean m_{inc} and standard deviation (SD) s_{inc} to be shared across all three distributions in the mixture model (*7–9*).

The likelihood for each candidate distribution $L_{inc}^{(l)}$ was defined as the product of the probability densities for each of the 13 imputed incubation periods:

3. $L_{inc}^{(l)} = \prod_{m=1,\ldots,13} f_l(s_m - e_m \mid \theta_{inc})$

where s_m and e_m are the imputed symptom onset and exposure times, respectively, and $f_l(\circ \mid \theta_{inc})$ is the probability density function (PDF) for the distribution l with the parameter set $\theta_{inc} = \{m_{inc}, s_{inc}\}$.

The likelihood for the mixture model, L_{inc}, was defined as the weighted sum of the individual likelihoods:

4. $L_{inc} = \sum_{l=1}^{3} w_l L_{inc}^{(l)}$

where w_l are the mixture weights ($\sum_l w_l = 1$). To compare the models, we calculated the posterior probability, P_l, that the entire dataset was generated by distribution l:

5. $P_l = \frac{w_l L_{inc}^{(l)}}{\sum_{u=1}^{3} w_u L_{inc}^{(u)}}$

The distribution with the highest posterior mean P_l is considered the best-fitting model for the data.

S1.3 Results

The mixture model identified the lognormal distribution as the best fit for the incubation period data (mean posterior weight: 72%), yielding a mean of 7.3 days (95% CI: 5.0–11.4) and an SD of 5.6 days (95% CI: 2.3–15.0) (**Appendix Figure 1**). The inferred distribution had a higher variance than anticipated, likely due to the small sample size. This finding contrasted with historical observations that incubation periods of 10 days or longer are rare (*4*). Therefore, we kept the mean at 7.3 days and reduced the SD to 2.0 days in our follow-up analysis for estimating the generation time. With these parameters, the 90th percentile of the distribution was 10.0 days.

S2 Outbreak data

Appendix Figure 2 depicts the data records of definitive transmission pairs collected during the school-based pertussis outbreak in South Korea, 2024 (*10*).

Each transmission pair i among total $N = 39$ records was composed of 1-day intervals of symptom onsets S_i: $[S_{L,i} = S_i, S_{R,i} = S_i + 1 \text{ day}]$. Adding a single day to the right boundary $S_{R,i}$ indicates that the interval spans from the midnight of $S_{L,i}$ to the midnight of the following day. The interdependency of the transmission pairs imposes that the symptom onset timing $S_{L,i} \leq s_i \leq S_{R,i}$ should be identical for the same unique ID. Let a subindex $k(j,i)$ map a transmission pair i of an infector $j = 1$ or infectee $j = 2$ to their unique ID number. Assuming that the symptom onset $s_{k(j,i)}$ is uniformly distributed within their interval, we wrote:

6. $s_{k(j,i)} \sim \text{Uniform}(\text{lower} = S_{k(j,i)}, \text{upper} = S_{k(j,i)} + 1 \text{ day})$

such that $s_{k(j',i')} \equiv s_{k(j'',i'')}$ for any $k(j',i') = k(j'',i'')$. Each transmission pair i thus possesses information on the inferred serial interval $s_{k(2,i)} - s_{k(1,i)}$, while the total number of unique IDs is $N_{\text{IDs}} = 42$.

S3 Imputation of infection times

First, we imposed priors on exposure times $e_{k(j,i)}$ $(j = 1,2; i = 1,2, \dots, N)$ of the infectors and infectees. If it is the index case, which implies that the case is a sole infector and not an infectee in any other transmission pairs, we impose:

7. $s_{k(1,i)} - e_{k(1,i)} \sim \text{HalfCauchy}(\text{mean} = 0, \text{SD} = 10)$

where the SD of 10 days represents a characteristic time scale of the incubation period. Otherwise, we imposed the uniform prior:

8. $e_{k(2,i)} \sim \text{Uniform}(\text{lower} = e_{k(1,i)}, \text{upper} = s_{k(2,i)})$

To account for the network structure of the transmission dynamics, we traversed the network starting from the index cases and then proceeding through secondary, tertiary, and subsequent cases.

We then require that the differences between the symptom onsets and exposure times follow a predetermined distribution of the incubation period:

$f(s_{k(j,i)} - e_{k(j,i)} \mid \theta_{inc} = \{m_{inc}, s_{inc}\})$

where f_{inc} is the probability density function (PDF) of the incubation period. As discussed above, the mean incubation period was set to 7.3 days and SD to 2.0 days. This allowed us to infer the posterior distribution of the exposure times $e_{k(j,i)}$ given the symptom onsets $s_{k(j,i)}$ and the incubation period distribution f_{inc}, expressed by the following likelihood:

9. $L_{inc,i} = \prod_{j=1,2} f_{inc}\left(s_{k(j,i)} - e_{k(j,i)} \,\middle|\, \theta_{inc}\right)$

Following the posteriors, we defined the generation time $e_{k(2,i)} - e_{k(1,i)}$, which follows a distribution $f_{gen}\left(e_{k(2,i)} - e_{k(1,i)} \,\middle|\, \theta_{gen} = \{m_{gen}, s_{gen}\}\right)$, where m_{gen} and s_{gen} are the mean and SD of the generation time. The serial interval can then be defined by the posterior:

10. $s_{k(2,i)} - s_{k(1,i)} = \left(e_{k(2,i)} - e_{k(1,i)}\right) + \left(s_{k(2,i)} - e_{k(2,i)}\right) - \left(s_{k(1,i)} - e_{k(1,i)}\right)$

where the right hand side is the generation time plus the difference between the incubation periods of the infectee and infector.

S4 Modeling of the generation time / serial interval (SI)

In our work we considered three standalone distributions for the generation time: gamma, Weibull, and lognormal, which are commonly used in the literature. Selection between them was performed using the mixture model within the Bayesian framework. Since the choice between distributions can be viewed as a categorical random variable, a common way of implementing this into the mathematical modeling is by using the mixture model. We defined the probability density function (PDF) of the generation time as $f_l\left(\theta_{gen}\right)$, and cumulative distribution function (CDF) as $F_l\left(\theta_{gen}\right)$, where $l = 1,2,3$ corresponds to one of the three distributions.

S4.1 Observed SI distribution

While estimating the observed serial interval or generation time distribution, we assumed no effect of the intervention on the collected data. While Cho *et al.* provided pointwise estimates of the serial interval, applying the maximum likelihood estimation (MLE) approach (*10*), we used a more flexible Bayesian framework accounting for transmission network structure and doubly-interval censored data (*11*). Because of this modification, our estimates will slightly differ from those of Cho *et al.*

S4.1.1 Generation time / SI

To estimate the generation time, we fitted the transmission pairs data to one of the three candidate distributions, either separately or within their mixture model. The latter approach allowed us to select the best-fitting distribution, as in Equations 4 and 5. For each candidate distribution, we defined the likelihood for the generation time as:

$$11.\ L_{gen}^{(l)}\left(\theta_{gen}\right) = \prod_{i=1,\dots,N} f_l\left(e_{k(2,i)} - e_{k(1,i)} \,\middle|\, \theta_{gen}\right)$$

where $e_{k(2,i)}$ and $e_{k(1,i)}$ are the exposure times of the infectee and infector, respectively, and θ_{gen} is the parameter set of the generation time distribution. These individual likelihoods were framed as a mixture model with relative weights ω_l:

$$12.\ L_{gen}\left(\theta_{gen} \,\middle|\, D\right) = \sum_{l=1}^{3} \omega_l L_{gen}^{(l)}\left(\theta_{gen}\right)$$

which is conditional on the data D. The best-fitting distribution was selected by the posterior probability:

$$13.\ \frac{\omega_l L_{gen}^{(l)}\left(\theta_{gen}\right)}{\sum_{u=1}^{3} \omega_u L_{gen}^{(u)}\left(\theta_{gen}\right)}$$

We imposed weakly informative priors on the parameters of the generation time distribution, such as the log-transformed mean and shape or squared coefficient of variation (CV), $\kappa 2_{gen}$. The prior for the log-transformed mean followed the normal distribution with the mean of 2.5 and SD of 5: $\log\left(m_{gen}\right) \sim \text{Normal}(\text{mean} = 2.5, \text{SD} = 5)$

If the Weibull distribution was part of the model, we imposed a prior on its log-transformed shape parameter as the standard normal distribution $\text{Normal}(\text{mean} = 0, \text{SD} = 1)$. Otherwise, we imposed a prior on the squared coefficient of variation (CV) of the generation time distribution as follows: $\kappa 2_{gen} \sim \text{gamma}(\text{shape} = 2, \text{scale} = 0.5)$

following recommendations of selecting prior distributions for Bayesian inference (*12*).

The serial interval was then derived as a posterior predictive distribution according to 10.

S4.1.2 Transmissibility

To quantify the transmissibility of pertussis, the number of secondary cases, n_k, generated by a primary case, k, was modeled with a negative binomial distribution (*10*). The

effective reproduction number, R_e, was defined as the mean of this distribution. The second parameter, which is an overdispersion parameter ϕ, quantified the superspreading potential. The likelihood for each case-patient k was then defined as:

14. $L_{p_0,k} = \text{NegBinomial}(n_k \,|\, \text{mean} = R_e,\ \text{overdisp.} = \phi)$

One additional case, $\tilde{k}$, with an unknown source of infection and no secondary cases, which occurred in the post-intervention period on May 10, was then added. In this case, the reproduction number was set to zero, $R_{\tilde{k}} = 0$, and the likelihood was given by:

15. $L_{p_0,\tilde{k}} = \text{NegBinomial}(0 \,|\, \text{mean} = R_e,\ \text{overdisp.} = \phi)$

S4.1.3 Likelihood

The total likelihood was then given by the product of one part for all case-patients k ($k \leq N_{IDs}$) and of another part for all transmission pairs i ($i \leq N$), which was framed as as a mixture model with relative weights ω_l:

16. $L_0(\Theta \,|\, D) = \sum_{l=1,2,3} \omega_l \prod_{k=1,\ldots,N_{IDs}} L_{inc,k} L_{p_0,k} L_{p_0,\tilde{k}} \prod_{i=1,\ldots,N} L_{gen,i}^{(l)}$

where $\Theta = \{\omega_l, \theta_{gen}, s_k, e_k, R_e, \phi\}$ is the parameter set of the model and D is the data. If a standalone distribution was used, the sum over l was omitted. The posterior probability of the model was then defined as:

17. $P_l = \frac{\omega_l \prod_i L_{gen,i}^{(l)}}{\sum_{u=1}^{3} \omega_u \prod_i L_{gen,i}^{(u)}}$

where the product over k was omitted because it does not depend on the distribution l.

S4.2 Unmitigated SI distribution

To infer the effectiveness of the intervention on 17 April 2024, we used the reconstructed network from Cho *et al.* (*10*). To quantify the effectiveness of intervention, we backprojected all cases to their date of infection (see the procedure above) and then segregated them into two groups: pre-intervention (before 17 Apr 2024) and post-intervention (after 17 Apr 2024). Among all cases, five were asymptomatic and excluded from this analysis. One case had an unknown source of infection and had no known secondary transmissions. Because their symptom onset was on 10 May 2024, we assigned them to the post-intervention period with case reproduction number set to zero.

The effect of intervention was modeled by two factors: shortening of the generation time / SI and reduction in transmissibility, resulting from reduced transmissibility due to intervention (**Appendix Figure 3** and **Appendix Figure 4**). We assumed that both factors had the same value ε ($0 \leq \varepsilon \leq 1$). We then inferred the unmitigated SI that would have expected in the counterfactual scenario of no intervention ($\varepsilon = 0$).

S4.2.1 Shortening of the generation time

We suppose that the main intervention at time t_{int} was implemented at noon on 17 Apr 2024. First, the intervention implied shortening of the generation time $e_{k(2,i)} - e_{k(1,i)}$ / serial interval $s_{k(2,i)} - s_{k(1,i)}$ by reducing the number of contacts of the infector. At the individual level, we modeled the effect of intervention by the factor ε. We distinguished three possible situations following the previous work of Chan and Nishiura (*13*):

1. When $e_{k(2,i)} > t_{int} > e_{k(1,i)}$, the likelihood for the generation time is defined as follows:

$$18.\ L_{gen,i}^{(l)} = \frac{(1-\varepsilon)f_l\left(e_{k(2,i)} - e_{k(1,i)} \,\middle|\, \theta_{gen}\right)}{(1-\varepsilon)\left(1-F_l\left(t_{int} - e_{k(1,i)} \,\middle|\, \theta_{gen}\right)\right)+F_l\left(t_{int} - e_{k(1,i)} \,\middle|\, \theta_{gen}\right)}$$

where the denominator is the normalization factor and $F_l\left(t_{int} \,\middle|\, \theta_{gen}\right)$ is the cumulative distribution function (CDF) of the generation time at t_{int}.

2. When $t_{int} > e_{k(2,i)}$ and $t_{int} > e_{k(1,i)}$, the likelihood is written as follows:

$$19.\ L_{gen,i}^{(l)} = \frac{f_l\left(e_{k(2,i)} - e_{k(1,i)} \,\middle|\, \theta_{gen}\right)}{(1-\varepsilon)\left(1-F_l\left(t_{int} - e_{k(1,i)} \,\middle|\, \theta_{gen}\right)\right)+F_l\left(t_{int} - e_{k(1,i)} \,\middle|\, \theta_{gen}\right)}$$

3. When $e_{k(1,i)} > t_{int}$, we get a simpler formula:

$$20.\ L_{gen,i}^{(l)} = f_l\left(e_{k(2,i)} - e_{k(1,i)} \,\middle|\, \theta_{gen}\right)$$

If the intervention is 100% effective, $\varepsilon = 1$, then only the situation 2 is realized. In this case, the right-truncated likelihood is obtained: $f\left(e_{k(2,i)} - e_{k(1,i)} \,\middle|\, \theta_{gen}\right)/F\left(t_{int} - e_{k(1,i)} \,\middle|\, \theta_{gen}\right)$.

S4.2.2 Reduction in transmissibility

Second, the intervention reduced the transmissibility of pertussis by reducing the contact rate between infector and potential infectees. To account for the effect of intervention, we

incorporated the offspring distribution—the distribution of secondary cases generated by a primary case—with a negative binomial distribution. The distribution is defined by two parameters: the mean, which is the reproduction number, and the overdispersion parameter, ϕ. Following earlier work (*13*), the case reproduction number in the pre-intervention period was defined as follows:

21. $R_k^{(l)} = R_e\left((1-\varepsilon)\left(1 - F_l\left(t_{int} - e_k \,\middle|\, \theta_{gen}\right)\right) + F_l\left(t_{int} - e_k \,\middle|\, \theta_{gen}\right)\right)$

while in the post-intervention period it was defined as:

22. $R_k^{(l)} = R_e(1-\varepsilon)$

The likelihood for the case k was then defined by the probability mass function of the negative binomial distribution:

23. $L_{p,k}^{(l)} = \text{NegBinomial}\left(n_k \,\middle|\, \text{mean} = R_k^{(l)}, \text{ overdisp.} = \phi\right)$

where n_k is the number of secondary cases reported for the case k. In addition to the 42 cases used in our analysis, we added one more case $\tilde{k}$ with an unknown source of infection and no secondary cases, that occurred in the post-intervention period on May 10. The reproduction number for this case was set to zero, $R_{\tilde{k}} = 0$, and the likelihood was given by:

24. $L_{p,\tilde{k}} = \text{NegBinomial}(0 \,|\, \text{mean} = R_e(1-\varepsilon), \text{ overdisp.} = \phi)$

S4.2.3 Likelihood

The overall likelihood was defined by the product of the likelihoods $L_{gen,i}$, $L_{p,k}$ and $L_{inc,k}$ for all transmission pairs i and all unique IDs k:

25. $L(\Theta \,|\, D) = \sum_{l=1,2,3} \omega_l \prod_{k=1 \ldots N_{IDs}} L_{inc,k} L_{p,k}^{(l)} \; L_{p,\tilde{k}} \prod_{i=1 \ldots N} L_{gen,i}^{(l)}$

which is conditional on the data D. $\Theta = \{\omega_l, \theta_{gen}, s_k, e_k, R_e, \varepsilon, \phi\}$ is the parameter set. Here, the likelihood $L_{inc,k}^{(l)}$ is also distribution-dependent as the time of exposures and symptom onsets are different for each distribution. The weights ω_l are the relative weights of the distributions, $\sum_{l=1}^{3} \omega_l = 1$. If a standalone distribution was used, the sum over l was omitted.

The probability of selection of the distribution l was defined by the expression:

26. $P_l = \frac{\omega_l \prod_k L_{p,k}^{(l)} \prod_i L_{gen,i}^{(l)}}{\sum_{u=1}^{3} \omega_u \prod_k L_{p,k}^{(u)} \prod_i L_{gen,i}^{(u)}}$

where the likelihoods $L_{inc,k}$ and $L_{p,\tilde{k}}$ were omitted because they do not depend on the distribution l.

We imposed weakly informative priors on the parameters following the published recommendations (*12*). First, the log-mean of the generation time was imposed with a normal prior: $\ln m_{gen} \sim \text{Normal}(\text{mean} = 2.5, \text{SD} = 5)$

Second, we distinguished two cases. When the Weibull distribution was used (e.g., as a part of the mixture model), we imposed the prior on the shape parameter of Weibull distribution with $\text{gamma}(\text{shape} = 2, \text{rate} = 0.5)$. Otherwise, we imposed the prior on the squared coefficient of variation (CV) of the generation time distribution: $\kappa 2_{gen} \equiv \left(\frac{s_{gen}}{m_{gen}}\right)^2 \sim \text{gamma}(\text{shape} = 2, \text{rate} = 0.5)$

The relative weights, exposure and symptom onset times were given with uninformative priors.

S4.3 Forward-looking reproduction number and generation time

Using the posterior distributions, we generated the posterior predictive distributions for the effective reproduction number following 21 and 22 and mean generation time as follows:

27. $R_e \left(F_l\left(t_{int} - t \,\middle|\, \theta_{gen}\right) + (1 - \varepsilon) \left(1 - F_l\left(t_{int} - t \,\middle|\, \theta_{gen}\right)\right)\right)$

for $t < t_{int}$ and $R_e(1 - \varepsilon)$ for $t \geq t_{int}$. The posterior predictive distribution for the mean generation time was defined as follows:

28. $\frac{(1-\varepsilon) m_{gen} + \varepsilon \int_0^{t_{int}-t} s f_l\left(s \,\middle|\, \theta_{gen}\right) ds}{(1-\varepsilon)\left(1 - F_l\left(t_{int} - t \,\middle|\, \theta_{gen}\right)\right) + F_l\left(t_{int} - t \,\middle|\, \theta_{gen}\right)}$

for $t < t_{int}$ and m_{gen} for $t \geq t_{int}$. The posterior predictive distribution for the mean SI would then be delayed by the incubation period.

The derived expressions 27 and 28 represent the 'forward-looking' parameters, which quantify the expected transmission potential of an individual infected at time t. We adopted this conceptual framework from Park *et al.* (Ref. 8 in the main text), as it correctly accounts for how

future interventions truncate the realized generation interval and reduce the effective reproduction number.

S5 Additional details on results

Appendix Figure 5 shows the posterior distribution of the generation times for the three candidate distributions (gamma, Weibull, and lognormal) and resulting mixture model. Results were consistent across all three candidate distributions. As anticipated, the unmitigated distribution was generally wider and had a larger mean and variance. Because we assumed the generation interval and incubation period were independent, the SI distribution had the same mean as the generation time distribution, but with larger variance (*14*). Notably, observing very long SIs in the range of 50-60 days appeared plausible, as the upper bound of the 95% CI approaches this range. Indeed, te Beest *et al.* reported a mean SI of 61.8 (95% CI: 40.5-97.1) days for father-to-sibling transmission pairs (*15*). However, such long intervals could also be prone to recall bias.

S6 Technical details

All analyses were undertaken in a Bayesian framework using Markov chain Monte-Carlo (MCMC) simulations, which allowed us to explore the space of parameters efficiently using the Metropolis-Hastings algorithm. Inference was performed using Stan software, version 2.36.0 (Stan Development Team, https://mc-stan.org). Each run of simulations consisted of 4 parallel chains with 15,000 posterior draws including 2,500 draws used for tuning and disregarded for the final output. The convergence of simulations was verified by ensuring the Gelman-Rubin R-hat statistic was below 1.01 (*16*).

All data and the computing code used to generate our results, are available at: https://github.com/aakhmetz/Pertussis-in-South-Korea-2024

S7 References

1. Rohani P, Scarpino S, editors. Pertussis: epidemiology, immunology, and evolution. Oxford: Oxford University Press; 2018. https://doi.org/10.1093/oso/9780198811879.001.0001

2. European Centre for Disease Prevention and Control. Systematic review on the incubation and infectiousness/shedding period of communicable diseases in children. 2016 [cited 2025 Aug 8]. https://www.ecdc.europa.eu/en/publications-data/systematic-review-incubation-and-infectiousnessshedding-period-communicable

3. Crowcroft NS, Pebody RG. Recent developments in pertussis. Lancet. 2006;367:1926–36. PubMed https://doi.org/10.1016/S0140-6736(06)68848-X

4. Gordon JE, Hood RI. Whooping cough and its epidemiological anomalies. Am J Med Sci. 1951;222:333–61. PubMed https://doi.org/10.1097/00000441-195109000-00011

5. Smith RE. A review of recent work on whooping-cough. QJM: An International Journal of Medicine. 1936;5:307–26. https://doi.org/10.1093/oxfordjournals.qjmed.a068007

6. Macdonald H, MacDonald EJ. Experimental pertussis. J Infect Dis. 1933;53:328–30. https://doi.org/10.1093/infdis/53.3.328

7. Keller M, Kamary K. Bayesian model averaging via mixture model estimation. arXiv. 2017:arXiv-1711. https://doi.org/10.48550/arXiv.1711.10016

8. Akhmetzhanov AR, Cheng HY, Linton NM, Ponce L, Jian SW, Lin HH. Transmission dynamics and effectiveness of control measures during COVID-19 surge, Taiwan, April-August 2021. Emerg Infect Dis. 2022;28:2051–9. PubMed https://doi.org/10.3201/eid2810.220456

9. Ponce L, Linton NM, Toh WH, Cheng HY, Thompson RN, Akhmetzhanov AR, et al. Incubation period and serial interval of mpox in 2022 global outbreak compared with historical estimates. Emerg Infect Dis. 2024;30:1173–81. PubMed https://doi.org/10.3201/eid3006.231095

10. Cho UJ, Cho S, Lee H, Kang SK, Kim BI, Nam Y, et al. Transmission dynamics and parameters for pertussis during school-based outbreak, South Korea, 2024. Emerg Infect Dis. 2025;31:1330–6. PubMed https://doi.org/10.3201/eid3107.241643

11. Reich NG, Lessler J, Cummings DA, Brookmeyer R. Estimating incubation period distributions with coarse data. Stat Med. 2009;28:2769–84. PubMed https://doi.org/10.1002/sim.3659

12. Stan Development Team. Prior choice recommendations. [cited 2025 Aug 8]. https://github.com/stan-dev/stan/wiki/prior-choice-recommendations

13. Chan YH, Nishiura H. Estimating the protective effect of case isolation with transmission tree reconstruction during the Ebola outbreak in Nigeria, 2014. J R Soc Interface. 2020;17:20200498. PubMed https://doi.org/10.1098/rsif.2020.0498

14. Lehtinen S, Ashcroft P, Bonhoeffer S. On the relationship between serial interval, infectiousness profile and generation time. J R Soc Interface. 2021;18:20200756. PubMed https://doi.org/10.1098/rsif.2020.0756

15. Te Beest DE, Henderson D, van der Maas NA, de Greeff SC, Wallinga J, Mooi FR, et al. Estimation of the serial interval of pertussis in Dutch households. Epidemics. 2014;7:1–6. PubMed https://doi.org/10.1016/j.epidem.2014.02.001

16. Gelman A, Rubin DB. Inference from iterative simulation using multiple sequences. Stat Sci. 1992;7:457–72. https://doi.org/10.1214/ss/1177011136

Appendix Table. Historical data collected on 13 pertussis cases with known incubation periods. The left and right bounds define the time interval (in days) for each recorded incubation period.

ID	Left bound, days	Right bound, days	Reference
Case 1	5	5	(5)
Case 2	4	4	(5)
Case 4	5	5	(5)
Case 6	2	3	(5)
Case 8	17	17	(5)
Case 11	7	9	(5)
Case 16	0	6	(5)
Case 20	4	4	(5)
Case 21	5	5	(5)
Case 22	6	6	(5)
Case 24	4	4	(5)
1	7	7	(6)
2	7	7	(6)

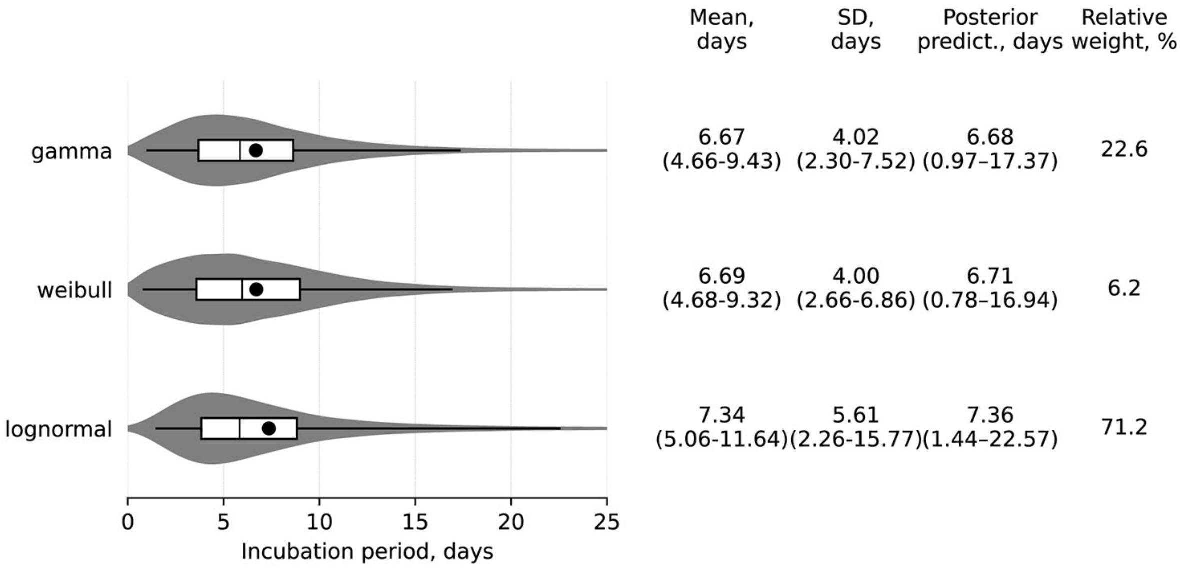

Appendix Figure 1. Comparison of estimated incubation period distributions (gamma, Weibull, lognormal) fitted to historical data (**Appendix Table**). Gray areas represent the posterior violin plot density, while each boxplot displays the interquartile range (box), 95% credible interval (whiskers), median (vertical line), and mean (solid circle). Summary statistics (mean, standard deviation [SD], posterior prediction), and relative weights are provided in the columns to the right.

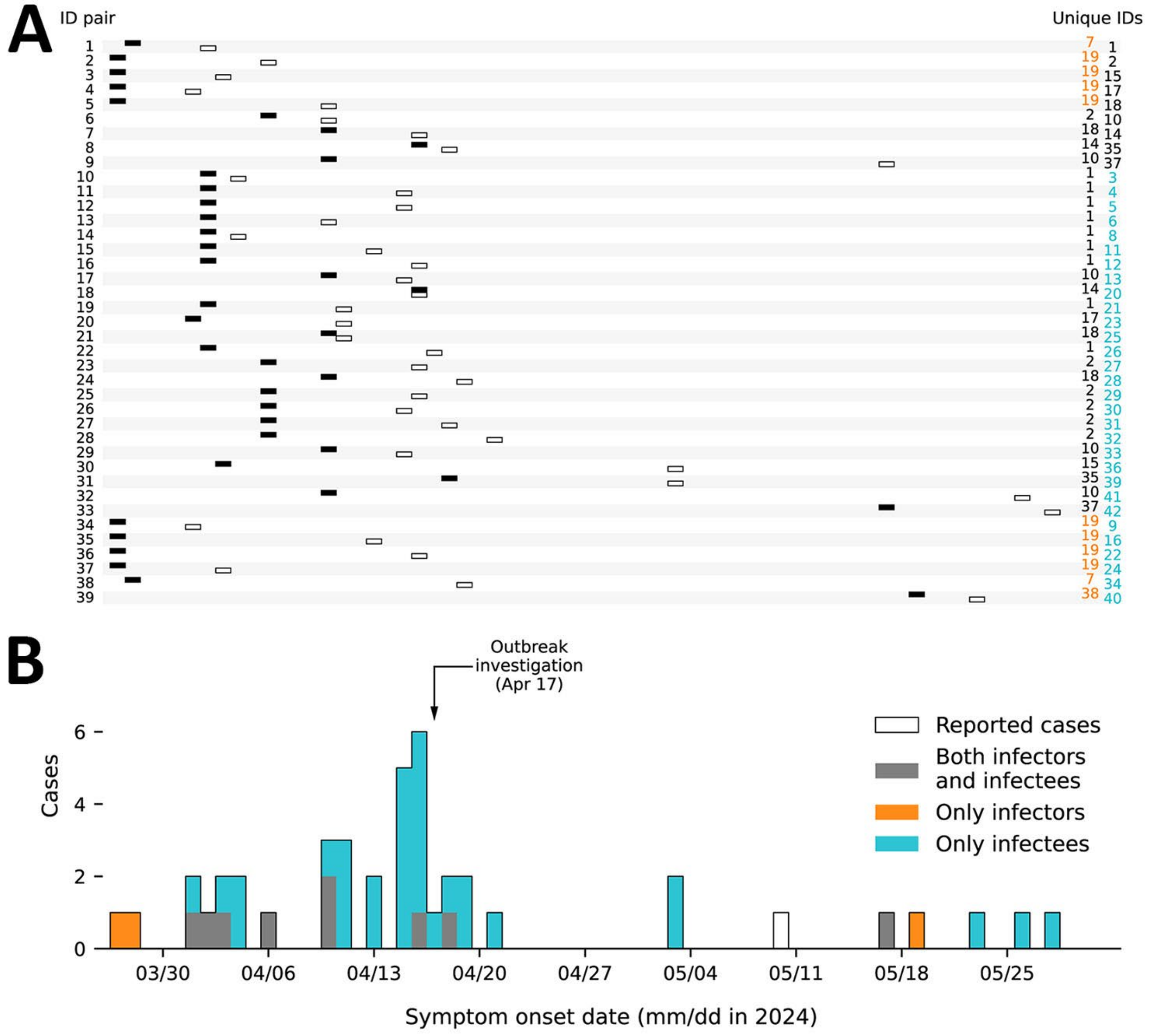

Appendix Figure 2. Transmission pairs and epidemic curve of the pertussis outbreak in South Korea, 2024. The top panel shows the timing of identified transmission pairs. Each row, distinguished by alternating background shading and labeled by pair ID on the left, shows the symptom onset of the infector (black rectangle) and the infectee (white rectangle). The right axis lists the case IDs for each pair: the infector in the left column and the infectee in the right column. Case IDs are color-coded by their role in the transmission: orange is for individuals who appear only as infectors, light blue is for those who appear only as infectees, and grey is for those recorded as both infectors and infectees. The bottom panel shows the epidemic curve by symptom onset date, with bars colored according to the same transmission roles. A sole white bar indicates a case that was not linked to any specific transmission pair. The arrow indicates the start of the outbreak investigation. The horizontal axis is shared among both panels.

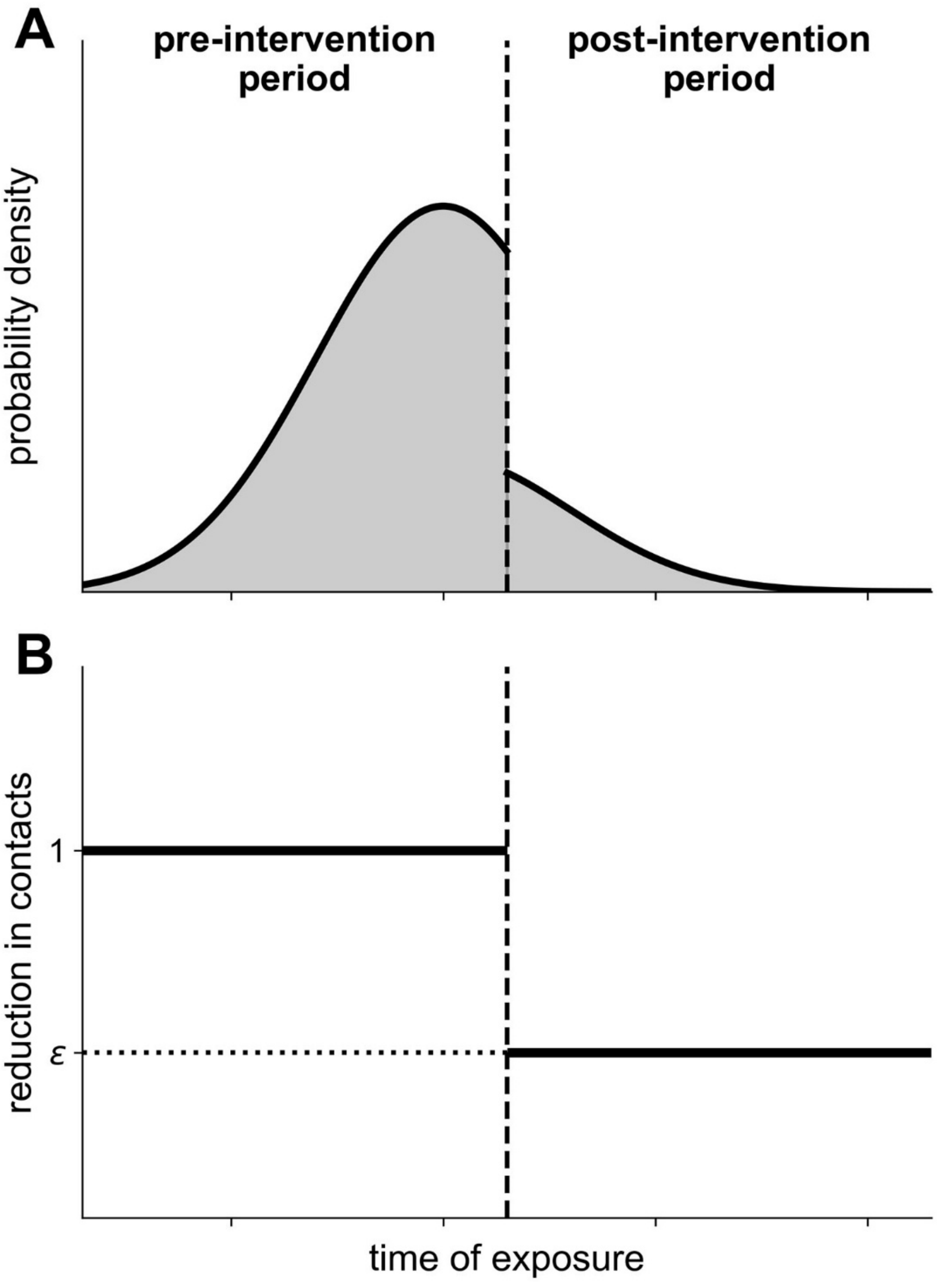

Appendix Figure 3. Conceptual diagram of a truncation-based modeling framework. (A) The probability density of the serial interval over time relatively to the major intervention (vertical dashed line). (B) The corresponding stepwise change in the reduction of contacts due to intervention (case-isolation). The relative contact rate is set to 1 (baseline) during the pre-intervention period and is reduced to a factor $\boldsymbol{\varepsilon}$ ($\mathbf{0} \le \boldsymbol{\varepsilon} \le \mathbf{1}$) during the post-intervention period.

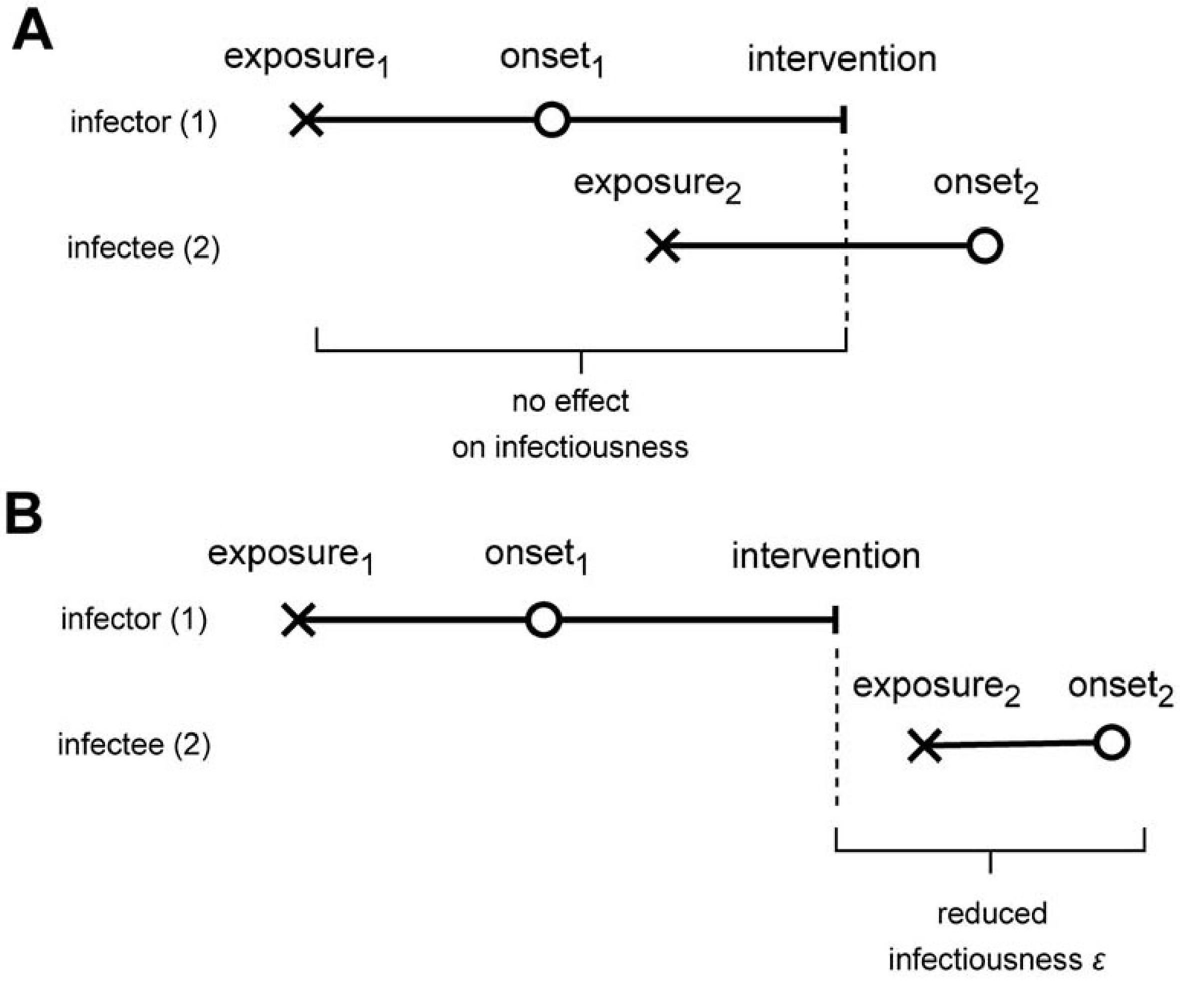

Appendix Figure 4. Classification of transmission events based on intervention timing. Illustration shows how transmission pairs are analyzed based on relative timing of the intervention (vertical dashed line). (A) Transmission occurs before the intervention, so there is no effect of intervention on transmissibility. (B) Transmission occurs after the intervention with reduced transmissibility to a factor $\boldsymbol{\varepsilon}$ ($\mathbf{0} \leq \boldsymbol{\varepsilon} \leq \mathbf{1}$), reflecting the effect of interventions. The infector is marked by index “1” and the infectee by index “2” . Crosses indicate the exposure times, and open circles indicate the symptom onsets.

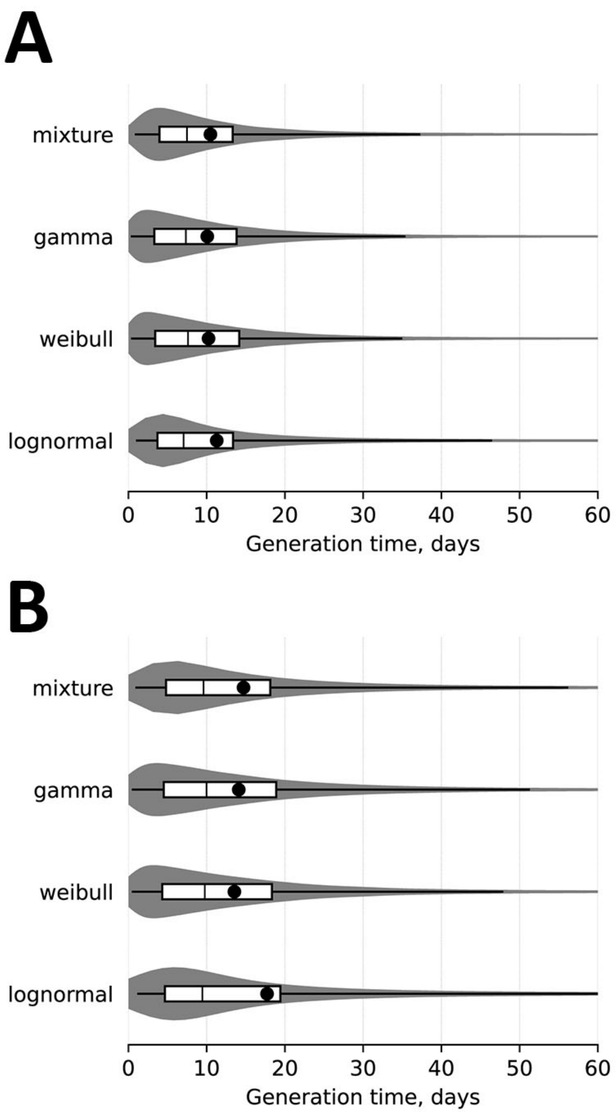

A

	Mean, days	SD, days	Posterior predict., days	Relative weight, %
mixture	10.45 (7.58-14.59)	10.42 (6.23-20.34)	10.49 (0.84–37.30)	–
gamma	10.12 (7.34-13.75)	9.28 (6.36-14.07)	10.08 (0.31–35.41)	21.0
weibull	10.21 (7.46-13.72)	9.22 (6.47-14.14)	10.25 (0.35–35.00)	16.0
lognormal	11.22 (7.73-16.78)	14.17 (7.02-30.42)	11.29 (0.98–46.48)	62.9

B

	Mean, days	SD, days	Posterior predict., days	Relative weight, %
mixture	14.74 (9.02-27.43)	16.72 (7.40-48.43)	14.72 (0.90–56.22)	–
gamma	14.07 (8.88-23.77)	13.13 (7.53-24.76)	14.11 (0.45–51.32)	25.2
weibull	13.55 (8.75-22.58)	12.44 (7.35-24.34)	13.55 (0.44–47.93)	23.6
lognormal	17.86 (9.62-36.41)	28.10 (9.31-82.19)	17.72 (1.13–82.77)	51.2

Appendix Figure 5. Comparison of observed (A) and unmitigated (B) generation time distributions estimated using a Bayesian mixture of gamma, Weibull, and lognormal distributions as well as their standalone versions. Gray areas represent the posterior violin plot density, while each boxplot displays the interquartile range (box), 95% credible interval (whiskers), median (vertical line), and mean (solid circle). Summary statistics (mean, standard deviation [SD], posterior prediction), and relative weights are provided in the columns to the right.
